# The Modulation of Melanogenesis in B16 Cells Upon Treatment with Plant Extracts and Isolated Plant Compounds

**DOI:** 10.3390/molecules27144360

**Published:** 2022-07-07

**Authors:** Anna Merecz-Sadowska, Przemysław Sitarek, Tomasz Kowalczyk, Karolina Zajdel, Ewa Kucharska, Radosław Zajdel

**Affiliations:** 1 Department of Computer Science in Economics, University of Lodz, 90-214 Lodz, Poland; radoslaw.zajdel@uni.lodz.pl; 2 Department of Biology and Pharmaceutical Botany, Medical University of Lodz, 90-151 Lodz, Poland; przemyslaw.sitarek@umed.lodz.pl; 3 Department of Molecular Biotechnology and Genetics, University of Lodz, 90-237 Lodz, Poland; tomasz.kowalczyk@biol.uni.lodz.pl; 4 Department of Medical Informatics and Statistics, Medical University of Lodz, 90-645 Lodz, Poland; karolina.smigiel@umed.lodz.pl; 5 Chair of Gerontology, Geriatrics and Social Work at the Faculty of Pedagogy, Ignatianum Academy in Cracow, 31-501 Cracow, Poland; ewa.kucharska@vadimed.com.pl

**Keywords:** melanoma cells, melanogenesis, signaling pathways, plant extracts, isolated plant compounds

## Abstract

Plants are a rich source of secondary metabolites that exhibit numerous desired properties. The compounds may influence the biology of melanocytes, pigment cells that produce melanin, by modulating numerous signaling pathways, including cAMP/PKA, MAPKs and PI3K/AKT. Its downstream target is microphthalmia-associated transcription factor, responsible for the expression of the tyrosinase enzyme, which plays a major role in melanogenesis. Therefore, this literature review aims to provide insights related to melanogenesis modulation mechanisms of plant extracts and isolated plant compounds in B16 cells. Database searches were conducted using online-based library search instruments from 2012 to 2022, such as NCBI-PubMed and Google Scholar. Upregulation or downregulation of signaling pathways by phytochemicals can influence skin hypo- and hyperpigmentation by changing the level of melanin production, which may pose a significant cosmetic issue. Therefore, plant extracts or isolated plant compounds may be used in the therapy of pigmentation disorders.

## 1. Introduction

Melanocytes, the melanin-producing cells, are located in the basal layer of the skin. Melanin accumulates in the melanosomes, which are then transferred to keratinocytes. The epidermal-melanin unit is composed of a single melanocyte and neighboring keratinocytes. Keratinocytes may also modulate melanocyte function and melanin production (melanogenesis): a complex biochemical process that begins with the transformation of the amino acid tyrosine by tyrosinase (TYR). The type and amount of melanin produced by melanocytes is genetically determined, but production is also influenced by many other internal and external factors, including ultraviolet (UV) radiation. The keratinocyte-derived paracrine factors, including melanocyte-stimulating hormone (α-MSH) and stem cell factor (SCF), may regulate melanocyte biology through receptor-mediated signaling pathways [1].

One of the major regulators of melanogenesis is microphthalmia-associated transcription factor (MITF), which is responsible for the activation of TYR expression. It is also the downstream target of many signaling pathways, including cyclic adenosine monophosphate (cAMP)/protein kinase A (PKA), mitogen-activated protein kinases (MAPKs), and phosphoinositide 3-kinases (PI3K)/protein kinase B (AKT). These pathways are induced in melanocytes by the activation of melanocortin 1 receptor (MCR1) and receptor tyrosine kinase (c-KIT) as a result of attachment of the corresponding ligand, i.e., α-MSH and SCF [2]. 

Plants are a rich source of secondary metabolites, which can be classified in various groups including phenolics, alkaloids, saponins, terpenes, lipids, and carbohydrates ac-cording to their chemical structure. It is estimated that more than 200,000 such molecules may exist in the plant kingdom [3]. Compounds of natural origin have several beneficial properties, including modulation of different signaling pathways. The pathways involved in the regulation of melanogenesis may also be subject to such modulation. That process can be both upregulated and downregulated [4]. Thus, owing to their properties plant extracts and isolated plant compounds may play a very important role in counteracting hyperpigmentation and hypopigmentation skin disorders [5]. 

This literature review examines the role of plant extracts and isolated plant compounds in the process of melanogenesis regulation in B16 cells, a well-established model for the discovery of melanogenic principles. The mechanisms of regulation are discussed in detail from the viewpoint of intracellular signal transduction pathways.

## 2. Study Design

Published data in the time range 2012–2022 were explored using widely-recognized databases such as NCBI-PubMed, Google Scholar, Scopus, and ScienceDirect. The following keywords were used: plant extract, plant-derived compound, melanogenesis, B16 cells, signaling pathways, ultraviolet radiation. This literature review included studies on plant extracts and pure compounds with available information on their modulatory effects on signaling pathways in B16 cells. Only compounds isolated directly from plants were included. Studies involving non-B16 cells, and synthetic compounds were excluded, as well as studies reporting only the final effects of extracts or compounds on melanogenesis without clarifying their molecular background. Items published in languages other than English or with only an abstract available were also rejected. In order to standardize the scientific names of plants, the “Medicinal Plant Names Services” (https://mpns.science.kew.org/mpns-portal/searchName?) (accessed on 10 May 2022) was used. PuBChem (https://pubchem.ncbi.nlm.nih.gov) (accessed on 10 May 2022) was used to obtain IUPAC names of pure compounds. 

## 3. Melanocyte Biology

While melanocytes are mostly found in the human skin, they can be also present elsewhere in the human body. In human skin, melanocytes are stationed in the basal layer of the epidermis and account for 1% of epidermal cells. The cells originate from neural crest cells, then mature and produce melanin in specialized organelles named melanosomes. The melanosomes are transferred to keratinocytes followed by pigment cell death. These cells express specific proteins including TYR, tyrosinase-related protein 1 and 2 (TYRP1, TYRP2) and MITF, among others. They form characteristic melanin units, in which one melanocyte is neighbored by 30–40 keratinocytes. Cross-talk exists between these two cell types. Keratinocytes influence the growth and activity of melanocytes through the action of growth factors and target adhesion molecules. Examples of compounds that are secreted by keratinocytes after UV radiation exposure and affect melanocytes include α-MSH, SCF, nerve growth factor (NGF), prostaglandin E2 (PGE2), endothelin (ET-1), granulocyte-macrophage colony-stimulating factor receptor (GM-CSFR) and basic fibroblast growth factor (bFGF). Melanocyte biology is also controlled by fibroblasts, which secrete SCF and neuregulin 1 (NRG1), among others. Upon stimulation, melanocytes also secrete a number of signaling molecules such as pro-inflammatory cytokines including IL-1α, IL-2, IL-3, IL-6, IL-10 and tumor necrosis factor α (TNF-α), as well as chemokines including IL-8, CCL2 and transforming growth factor (TGF-β), α-MSH, catecholamines, eicosanoids, serotonin and nitric oxide (NO) [1,6,7].

**Figure 1:**
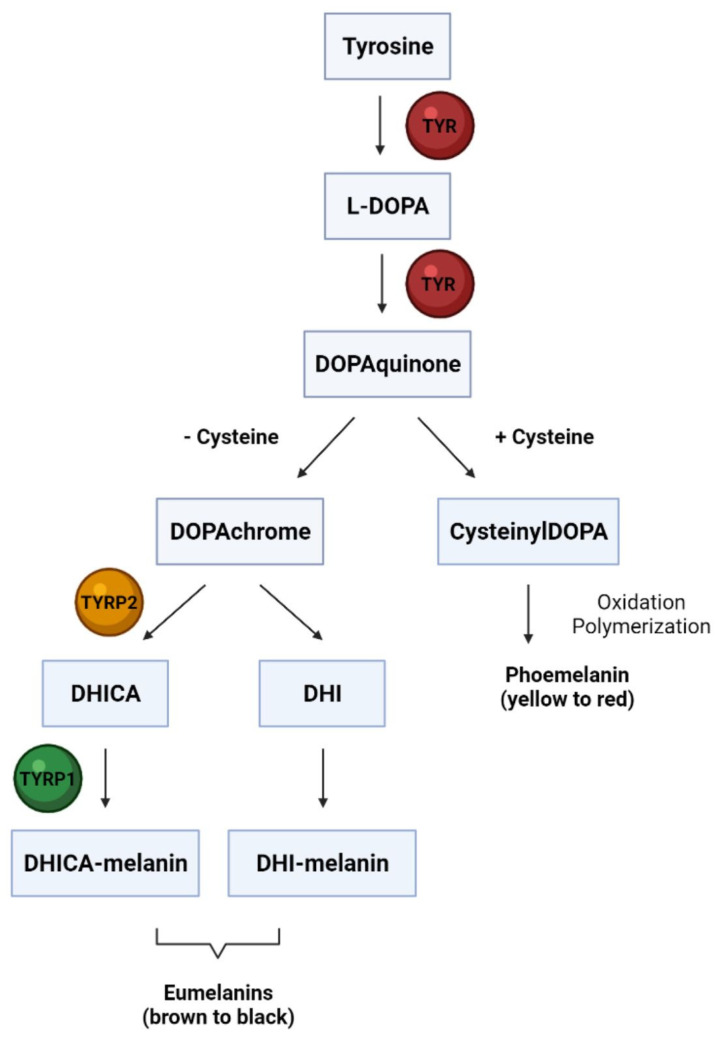
Schematic presentation of melanin synthesis during melanogenesis in melanocytes. [Created by BioRender].

Melanocytes synthesize melanin through a biochemical pathway called melanogenesis. The process takes place in separate cytoplasmic organelles called melanosomes. Melanin is typically found as yellow-–red pheomelanin and dark brown–black eumelanin, whose production is determined by enzyme action and substrate availability. TYR catalyzes the hydroxylation of tyrosine to L-3,4-dihydroxyphenylalanine (DOPA). DOPA is then oxidized to DOPAquinone. DOPAquinone in the presence of cysteine leads to the formation of 3- or 5-cysteinylDOPAs, which then yield pheomelanin by conversion. In the absence of thiol substrates, DOPAquinone undergoes cyclization to DOPAchrome. DOPAchrome leads to the formation of 5,6-dihydroxyindole, which upon transformation forms eumelanin. However, in the presence of DOPAchrome tautomerase (TYRP2), 5,6-dihydroxyindole-2-carboxylic acid is formed from DOPAchrome. Further transformations involving TYR and TYRP1 finally produce the brown color of melanin (Figure 1). 

Human skin contains a mixture of all types of melanin, and visible pigmentation is determined by their ratio. In addition, melanin has numerous properties that are beneficial to the body; while the most important one is the absorbance and scattering of UV radiation, the molecule also plays a part in the neutralization of free radicals [4].

**Figure 2:**
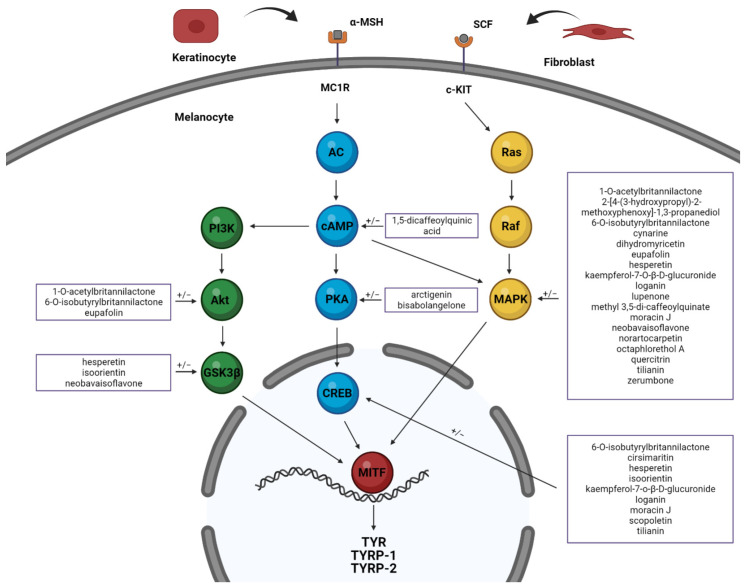
Selected signaling pathways involved in melanogenesis that are modulated by several isolated plant compounds. [Created by BioRender].

Melanogenesis is regulated by more than 125 genes [8]. Many factors, including external agents (UV radiation), and both internal and paracrine factors produced by keratinocytes and fibroblasts, stimulate specific intracellular signaling pathways involved in melanogenesis, including cAMP/PKA, MAPKs and PI3K/AKT. The most important regulator of melanogenesis is MITF. It affects the activation of key melanogenesis-related genes such as TYR, TYRP1 and TYRP2, and is a common downstream target of many signal transduction pathways that may be modulated by plant extracts and isolated plant compounds (Figure 2) [2]. 

For terminally differentiated cells, their proliferation is in turn inhibited [9]. Despite their very low proliferative capacity, melanocytes undergo telomerase-dependent senescence. Senescence results in characteristic morphological and functional changes. This is an irreversible process and cells cannot be stimulated to proliferate again by agents such as growth factors. A crucial role in senescence is played by the retinoblastoma protein (pRb). It acts as a repressor of genes involved in DNA replication, resulting in cell cycle arrest. Along with pRb, other cell cycle inhibitors such as p16INK4a and p21Waf1 and their homologs interact to inhibit cyclin-dependent kinases (CDKs) activity and thus prevent phosphorylation of pRB, keeping it in an activated state [10]. For example, *Spartium junceum* flowers inhibit melanogenesis in B16 melanoma cells by inducing senescence caused by cell cycle arrest in the G2/M phase [11].

Furthermore, it has been shown that melanocytes can also undergo stress-induced senescence. UVB radiation exposure was confirmed to affect melanocyte proliferation. Exposure to low and repeated doses of UVB radiation increased β-galactosidase activity associated with senescence, and changes in the expression of other markers, including p21, p53 and lamin B1. In addition, UVB radiation was shown to contribute to impairment of the proteasome, intensification of autophagy processes in melanocytes, and increased intracellular melanin levels. For example, autophagy may also be induced by hydroxydaidzein from fermented soybean paste [12]. Aging melanocytes accumulating in the skin are also thought to impair the proliferation of neighboring keratinocytes [13,14].

## 4. Natural Skin Agents against Hyper and Hypo-Pigmentation Disease

Alterations in skin pigmentation might become an esthetic problem. Common hyperpigmentation disorders include melasma, solar lentigines, post-inflammatory hyperpigmentation, and chloasma. UV radiation exposure can exacerbate all of these conditions. Clinically, it manifests as a brown or blue discoloration of the skin, whose location depends on the site of melanin deposition, i.e., in the epidermis and dermis. Hypopigmentation disorders include vitiligo which is related to genetic and environmental factors. Vitiligo is clinically manifested by the presence of white patches on the skin; the loss of melanocyte activity in these areas is probably the result of melanocyte destruction [5]. Plants serve as a reservoir for ingredients that can be used to improve the appearance of the skin. Metabolites of plant origin are classified as primary and secondary based on their role. The former are involved in the basic physiological functions of the cell, while the latter are intended to provide defense against adverse environmental conditions, herbivores, and pathogens [15].

Secondary metabolites can be divided into three main groups depending on the pathway of their synthesis: terpenes formed via the mevalonate pathway, phenolic com-pounds formed via the szikimic or mevalonate pathway, nitrogen-containing secondary metabolites formed mainly from aliphatic amino acids via the tricarboxylic acid pathway, and aromatic acids derived from the szikimic acid pathway [16]. Due to their chemical structure, secondary metabolites are divided into phenolics, alkaloids, saponins, terpenes, lipids or carbohydrates [17].

In the search for new depigmenting and pigmenting agents, studies of plant extracts have led to the identification of many potentially active compounds. Depigmenting agents may act at different levels of melanin production, many are activators of tyrosinase, a key enzyme involved in melanogenesis and pigment production. Others may affect the expression of this enzyme, in addition to the transport of pigment from melanocytes to keratinocytes. On the other hand, due to the lack of pigment cells, attempts have been made to find compounds that influence the differentiation and migration of melanoblasts or would enable melanin dispersion and induce skin pigmentation.

A great number of plant extracts and isolated plant compounds have been found to bear adequate anti-melanogenic or melanogenic potential. They may even have an additional protective potential due to their antioxidant properties, thanks to which they protect the cell’s macromolecules from the damaging effects of free radicals generated by UV radiation, among others. In conclusion, plant-derived compounds may form part of therapeutic interventions against skin abnormalities, including hyperpigmentation and hypopigmentation [18,19,20,21,22].

## 5. Mechanisms of Melanogenesis-Related Signaling Pathway Modulation by Plant Extracts and Isolated Compounds in B16 Cells

Many genes that encode diverse proteins are implicated in different steps of pigmentation such as melanocyte formation from the neural crest, formation of melanosome components, pigment inclusion, and the transfer of melanosomes from melanocytes to keratinocytes [23]. Plant extracts and isolated plant compounds may be able to modulate pigment formation. Most previous studies are based simply on the assessment of melanin content and tyrosinase enzyme activity in melanocytes exposed to phytochemicals. Others examine changes in gene expression and protein production that are particularly important for melanogenesis. These include MITF, a master regulator of melanogenesis gene expression; this activates the genes encoding the pigmentation enzyme TYR and tyrosinase-related proteins (TYRPs) by binding to their promotors. Data on their expression and its modulation by plant extracts without and with identified compounds (Table 1 and Table 2) and single-derived compounds (Table 3) are presented. Additionally, the roles of phytochemicals in various signaling pathways are also analyzed. Activation of melanocortin 1 receptor (MC1R) or receptor tyrosine kinase (c-KIT) by α-MSH or SCF ligands, respectively, activate signaling pathways in melanoma cells that may be modulated by plant extract or isolated plant compounds. Natural chemicals may have the effect of both stimulating and inhibiting melanogenesis. Global expression analysis by Villareal et al. identified MITF modulation in B16 melanoma cells treated with the Cymbopogon schoenanthus extract, as well as 44 other pigmentation-related genes [24].

### 5.1. cAMP/PKA Signaling Pathway

The neuromodulating peptide α-MSH is released by keratinocytes after stimulation with pro-inflammatory cytokines or UV light. The junction of ligand to MCR1 located on the melanocyte cell surface activates adenylyl cyclase, responsible for the synthesis of cAMP. Increased cAMP levels cause stimulation of PKA. Both the cAMP level and melanin production are increased following treatment of B16 melanoma cells with 1,5-dicaffeoylquinic acid isolated from *Vernonia anthelmintica* seeds [93]. *Phyllostachys nigra* stem extracts were found to have an anti-melanogenic effect in B16 melanoma cells by decreasing intracellular cAMP and PKA levels [94], as did *Dendropanax morbiferus* leaves [95] and *Lotus seedpod* extract [96]. Among single-derived compounds, bisabolangelone, a sesquiterpene derivative, isolated from *Angelica koreana* roots [97] and arctigenin, a lignan, from *Arctium lappa* seeds inhibit cAMP-dependent PKA activation [98]. 

PKA entails phosphorylation at Ser133 of cAMP-response element binding protein (CREB) and subsequent activation of MITF expression. The extracts that up-regulate pigment formation are *Melia azedarach* bark [99] and *Dalbergia odorifera* fruit [100]. In B16 melanoma cells, they increase the intracellular cAMP level and PKA activity, translating to increased phosphorylation of CREB, its downstream signaling protein, followed by up-regulation of MITF and TYR expression. However, no effects on MAPKs were observed. Moreover, CREB was activated by cirsimaritin, a dimethoxyflavone, isolated from the branches of *Lithocarpus dealbatus* [101], hesperetin, a flavanone glycoside, from *Citrus sinensis*, *Citrus aurantium* and *Citrus reticulata* extract [102] and scopoletin, a hydroxycoumarin, from the aerial parts of *Cirsium setidens* [103]. The opposite effect on phosphorylation of PKA and CREB are interfered with by *Nelumbo nucifera* leaf extract [104]. *Rhodiola rosea* root extract [105], *Elaeagnus umbellate* branches and leaves [106], *Oenothera laciniata* extracts [107] and *Kadsura coccinea* roots, stems, leaves, fruits [108] extracts inhibited the phosphorylation of CREB followed by downregulation of MITF and TYR expression. Similar inhibitory effects on CREB exerts isoorientin, a flavone glycoside, derived from *Gentiana veitchiorum* flowers [109], polysaccharide from *Morchella esculenta* fruits [110], loganin, an iridoid monoterpenoid, from *Cornus officinalis* [111], kaempferol-7-O-β-D-glucuronide, flavonol glucoside, and tilianin, a flavonoid glycoside, isolated from aerial parts *Cryptotaenia japonica* [112], moracin J, a 2-arylbenzofuran flavonoid, from leaves of *Morus alba* [113], and 6-O-isobutyrylbritannilactone from the flowers of *Inula britannica* [114]. This pathway is also suppressed by *Phoenix dactylifera* seed extract that decreased the expression of MC1R [115].

### 5.2. MAPKs Signaling Pathway

Both α-MSH-MC1R and SCF-c-KIT junction may modulate the MAPKs pathway. MAPKs regulate cell proliferation, differentiation, motility and survival by converting extracellular signals into intracellular cellular responses. The best-studied Ser/Thr kinases in this family are extracellular signal-regulated kinases 1 and 2 (ERK1/2), c-Jun amino-terminal kinases 1–3 (JNK 1 to -3), and p38 (α, β, γ, and δ). Each of these groups consists of a set of three sequentially acting kinases: MAPK, MAPK kinase (MAPKK) and MAPKK kinase (MAPKKK). Activation of MAPKKK leads to phosphorylation and activation of MAPKK, which in turn activates MAPK through dual phosphorylation of Thr residues within the Thr-X-Thr motif located in the activation loop of the kinase domain [116]. 

It is assumed that the increased level of cAMP triggered by MC1R activation is followed by induction of ERK in a cell-type seemed to be mediated by B-RAF, such as melanoma cells [117]. cAMP levels seemed to have the opposite effect on p38 activity. The effect of cAMP on the JNK signaling cascades has been less explored; however, in the majority of cells, increased cAMP levels appeared to modulate JNK activity [118].

SCF is a growth factor secreted by human keratinocytes and fibroblasts under UV radiation [119,120]. It is believed to be involved in the dimerization and autophosphorylation of the c-KIT receptor by binding to the extracellular domain, thus activating it. Phosphorylation at Y703 and Y936 induce MAPKs. Following activation, the c-KIT phosphorylates the small GTPase (Ras), which in turn induces proto-oncogene serine/threonine-protein kinase (Raf-1) followed by the MAPKs signaling pathway [121].

Activated p38 induces melanogenesis via CREB phosphorylation followed by MITF activation and TYR expression. Activation of ERK phosphorylates MITF at Ser73, leading to its degradation via ubiquitination and thus inhibiting melanogenesis. JNK activation may modulate melanogenesis through phosphorylation of CREB-regulated transcription co-activator 3 (CRTC3)-dependent MITF expression [122,123].

P38 positively regulates melanin synthesis in the B16 melanoma cell line [2]. In this line, *Polygonum multiflorum* root extract was found to up-regulate p38 phosphorylation, thus activating melanogenesis [124]; the same effects were observed for *Vernonia anthelmintica* seed extract [125] and *Annona squamosa* leaf extract [126]. In contrast, treatment with *Cuscuta japonica* seed extract [127], *Morinda citrifolia* fruit and leaf extract [128], *Dendropanax morbiferus* leaf extract [95] or *Lotus seedpod* extract [96] were found to downregulate p38 phosphorylation and thus inhibit melanogenesis. Similar effects are demonstrated by the polysaccharide from *Morchella esculenta* fruits [110], methyl 3,5-di-caffeoylquinate from the stems and leaves of *Erigeron annuus* [129] and moracin J, a 2-arylbenzofuran flavonoid, from *Morus alba* leaves [113]. 

ERK negatively regulates melanin synthesis in the B16 melanoma cell line. ERK may be suppressed by *Ardisia crenata* leaf and small branch extract [130] and by lupenone, a triterpenoid, isolated from *Erica multiflora* leaf [131]. In contrast, ERK may be upregulated, and melanogenesis suppressed, by *Astragalus membranaceus* whole plant extract [132], *Aster yomena* callus pellet extract [133], *Melochia corchorifolia* whole plant extract [134], *Artemisia capillaris* whole plant extract [135], *Orostachys japonicus* whole plant extract [136], and *Aster spathulifolius* leaf extract treatment [137]. Similar effects are also triggered by 2-[4-(3-hydroxypropyl)-2-methoxyphenoxy]-1,3-propanediol isolated from *Juglans mandshurica* fruits [138], 1-O-acetylbritannilactone from *Inula britannica* flowers [139], zerumbone, a sesquiterpenoid, from *Zingiber officinale* [140], octaphlorethol A, a phenolic compound, from *Ishige foliacea* [141], 2-[4-(3-hydroxypropyl)-2-methoxyphenoxy]-1,3-propanediol from *Juglans mandshurica* fruits [138], loganin, an iridoid monoterpenoid, from *Cornus officinalis* [111], neobavaisoflavone, a 7-hydroxyisoflavone, from aerial parts of *Pueraria lobata* [142], 6-O-isobutyrylbritannilactone from *Inula britannica* flowers [128] and quercitrin, a quercetin O-glycoside, from *Lindera obtusiloba* leaves [143]. 

*Arctium lappa* leaf extract was found to inhibit melanogenesis in B16 melanoma cells by regulating JNK phosphorylation [144].

Many studies have examined the influence of plant extracts and isolated compounds on various MAPKs in B16 melanoma cells. Extracts derived from *Artocarpus communis* heartwood [145], *Phyla nodiflora* aerial part [146] and *Oenothera laciniata* [107] were found to modulate ERK and JNK phosphorylation, resulting in anti-melanogenic effects. Similar results were obtained for kaempferol-7-O-β-D-glucuronide and tilianin, a flavonoid glycoside isolated from the aerial parts of *Cryptotaenia japonica* [126]. *Penthorum chinense* whole plant extract [147], *Kaempferia galanga* whole plant extract [148] and *Phragmites communis* leaf extract [149] were found to modulate both p38 and JNK phosphorylation, followed by reduced melanin production, in B16 melanoma cells. This phosphorylation was also modulated by norartocarpetin, a flavone, obtained from *Artocarpus communis* heartwood [150]. *Argania spinosa* fruit extract [151] upregulates melanogenesis through the modulation of p38 and ERK signaling. Among single-derived compounds this effect was obtained for cynarine, a hydroxycinnamic acid derivative, extracted from *Vernonia anthelmintica* [93] and hesperetin, a flavanone glycoside, from *Citrus sinensis*, *Citrus aurantium* and *Citrus reticulata* extract [102]. Eupafolin, a flavone, isolated from the aerial part of *Phyla nodiflora* was found to suppress melanogenesis via p38 and ERK modulation in B16 cells [152]. *Paederia foetida* whole plant extract was found to modulate the activity of all MAPKs, resulting in inhibited melanin production [153]; this was also observed for *Rosa gallica* petal extract [154] as well as dihydromyricetin, a flavanonol, isolated from the leaves and stems of *Ampelopsis grossedentata* [155].

### 5.3. PI3K/AKT Signaling Pathway

MC1R activated by α-MSH, as well as c-KIT activated by SCF, can also modulate the PI3K/AKT pathway: a very important process regulating cell proliferation and survival. It has been shown that MC1R activation followed by induction of cAMP leads to the inhibition of PI3K activity and of AKT phosphorylation and activity. Inactive AKT is unable to phosphorylate glycogen synthase kinase 3 beta (GSK3β) [156,157,158]; this phosphorylation is needed for β-catenin accumulation, followed by translocation to the nucleus. This in turn stimulates MITF activity and TYR gene expression. SCF is believed to be involved in the dimerization and autophosphorylation of the c-KIT receptor by binding to the extracellular domain, thus activating it, phosphorylation at Y721 and PI3K pathway induction. Activated AKT phosphorylate GSK3β becomes inactivated and targeted for proteasomal degradation [121,159]. 

In B16 melanoma cells, *Musa sapientum* peel extract [160], *Aster yomena* callus pellet extract [133] and *Orostachys japonicus* whole plant [136] treatment reduced phosphorylation of AKT, and these might act as anti-melanogenesis agents. Upregulation of AKT by *Phragmites communis* leaf extract [149], *Aster spathulifolius* leaf extract treatment [137] and *Ginkgo biloba* leaf extract [161] may also inhibit melanin synthesis. Among plant-derived compounds, 1-O-acetylbritannilactone obtained from *Inula britannica* flowers [139], eupafolin, a flavone, isolated from aerial part of *Phyla nodiflora* [152] and 6-O-isobutyrylbritannilactone from the flowers of *Inula britannica* have been found to increase AKT signaling in B16 cells [114]. GSK3β activation is stimulated by hesperetin, a flavanone glycoside, from *Citrus sinensis*, *Citrus aurantium* and *Citrus reticulata* extract [102], an agent with melanogenic potential; and is also inactivated by isoorientin, a flavone glycoside, derived from *Gentiana veitchiorum* flowers, and neobavaisoflavone, a 7-hydroxyisoflavone, from aerial parts of *Pueraria lobata* [142], agents with whitening potential [109].

### 5.4. In Vivo Studies

In some studies, given above, in vivo analyses were also conducted in addition to the in vitro study and evaluation of signaling pathway modulation by plant extracts and compounds isolated from plants. The melanocyte activity and the distribution of melanin granules were decreased in UVB-irradiated C57BL/6 mice treated with *Aster spathulifolius* leaf extract. Mice were exposed to UVB radiation at a dose of 100 mJ/cm^2^ for 10 days. Extract was orally administered for 9 weeks at 35, 70 and 140 mg/kg concentrations [137]. *Nelumbo nucifera* leaf extract reduced skin melanogenesis induced by UVB radiation in guinea pigs. The animals were exposed to UVB radiation three times a week for two weeks. The total UVB dose was 500 mJ/cm^2^ per exposure. Then, 1 or 2% of extract was given topically to the UVB-irradiated regions the next day [96]. Treatment of subjects with skin pigmentation with *Aster yomena* callus pellet extract-containing cream formulations resulted in 3.33%, 7.06%, and 8.68% improvements in melanin levels at 2, 4, and 8 weeks, respectively. These results suggest that *Aster spathulifolius, Nelumbo nucifera and Aster yomena* extracts might be vulnerable and promising therapeutics as agents for hyperpigmentation [133].

Studies based on single compounds indicate that arctigenin from *Fructus arctii* demonstrated antimelanogenic activity using zebrafish embryo. Arctigenin was added directly in fish water at final concentrations of 1, 10, and 100 𝜇M [98]. The same result was obtained for the zebrafish embryo treated with 10, 50, or 100 µM 6-O-isobutyrylbritannilactone from *Inula britannica* [114]. In addition, a similar result was also observed for the zebrafish embryo treated with 75, 150 and 300 μg/mL heteropolysaccharide from *Morchella esculenta* [110]. The embryos had significantly reduced pigmentation in the arctigenin, 6-O-isobutyrylbritannilactone and heteropolysaccharide-treated specimens. In conclusion, the results suggest that these compounds have potential as a skin-whitening agent for hyperpigmentation.

## 6. Mechanisms of Melanogenesis-Related Signaling Pathway Modulation by Plant Extracts and Single-Derived Compounds in B16 Cells Stimulated by UV Radiation

Exposure to UV radiation has deleterious effects on human skin followed by acute and chronic skin damage. It also generates the production of ROS and proinflammatory cytokines. Both experimental and epidemiological data indicate that the melanin present in skin plays an important role in photoprotection.

UV radiation interferes directly with macromolecules, including proteins, lipids and nucleic acids. Interactions with nucleic acids in skin cells may contribute to DNA damage. If these mutations occur in genes responsible for the regulation of repair processes, cell cycle or apoptosis, they may subsequently initiate oncogenic transformation.

UVA can generate ROS, which are capable of inducing oxidative DNA damage: singlet oxygen activity and type 1 photosensitization reactions result in oxidative modifications of nitrogenous bases, mainly guanine. If not repaired, 7,8-dihydro-8-oxoguanine lesions are formed leading to mutations. The major mutations are the G -> A transition and the G -> T transversion. Cyclobutanopyrimidine dimers (CPDs) may also form.

UVB leads to the formation of photoproducts such as CPDs and pyrimidine 6-4 pyrimidones in DNA due to the activation of a photochemical reaction, usually occurring between adjacent pyrimidine nucleotides. If left unrepaired, they can lead to mutations, including C -> T and CC -> TT transition mutations, and oncogenesis [162].

UV radiation exposure induces DNA damage in keratinocytes and activates the p53 tumor suppressor protein, which can bind to and activate the pro-opiomelanocortin (POMC) promoter. It can also induce production of subunit melanogenic peptides, including α-MSH which binds to the MCR1 on melanocytes, thus stimulating the expression of genes involved in melanin production [163]. 

Certain plant extracts and isolated plant compounds are able to inhibit UV-induced melanogenesis, as observed in stimulated B16 melanoma cells. *Psidium guajava* leaf [164] and *Foeniculum vulgare* fruit [165] extracts block the activity of TYR and calcium release-activated calcium channel protein 1 (ORAI1). The ORAI1 channel is also inhibited by valencene, a sesquiterpene, isolated from the rhizomes of *Cyperus rotundus* [166]. 

UV radiation directly induces intracellular calcium signaling in melanocytes, mediated in part by the ORAI1 channel. In addition, UV radiation stimulates melanocytes through compounds such as endothelin 1 (ET-1) released by keratinocytes. Activation of the ET-1 receptor also triggers intracellular calcium signaling, mediated by ORAI-1. Increased calcium levels activate TYR, resulting in melanin production. Therefore, ORAC channel antagonists play a key role in inhibiting UV-induced melanogenesis [167,168].

## 7. Conclusions

Plant extracts or isolated plant compounds, including in particular phenolics and terpenes may act as activators or inhibitors of key signaling pathways, such as cAMP/PKA, MAPKs and PI3K/AKT in melanocytes. Such modulation influences the expression of proteins, including master regulator of melanogenesis-MITF. Therefore, natural chemicals may serve as useful ingredients for reducing skin pigmentation or activating pigment formation. However, despite the very high potential of plant-derived molecules, proposals for the future work include improved exploration of the signaling pathways that may be modulated by phytochemicals in the melanogenesis process as well as better evaluation of their effects on living organisms.

## Figures and Tables

**Table 1:** Modulation on gene expression related to melanogenesis in B16 melanoma cells by plant extracts without identified compounds.

Name of Species/Family	Part of Plant	Type of Solvent	Concentration	Methods	Effects	Ref.
*Artemisia asiatica* Nakai ex Pamp./Asteraceae	whole plant	ethanol	25–50 µg/mL	RT-PCR, Western blotting	Reduced expression: MITF, TYR, TYRP-1, TYRP-2	[25]
*Camellia sinensis* (L.) Kuntze/Theaceae	flower	ethanol	20–40 µg/mL	RT-PCR	Reduced expression: TYR	[26]
*Castanea crenata* Siebold & Zucc./Fagaceae	inner skin	ethyl acetate	10–100 µg/mL	Western blotting	Reduced expression: TYR	[27]
*Cinnamomum osmophloeum* Kaneh./Lauraceae	leaves	ethanol	21.25 µg/mL	RT-PCR	Reduced expression: MITF, TYR	[28]
*Coix lacryma-jobi* L./Poaceae	seeds	ethanol	20–40 mg/mL	RT-PCR, Western blotting	Reduced expression: MITF, TYR, TYRP-1, TYRP-2	[29]
*Croton roxburghii* N.P.Balakr. and Croton sublyratus Kurz/Euphorbiaceae	leaves	ethanol	25–100 µg/mL	RT-PCR, Western blotting	Reduced expression: MITF, TYR, TYRP-1, TYRP-2	[30]
*Dendrobium moniliforme* (L.) Sw./Orchidaceae	leaves	ethanol	12.5–50 µg/mL	Western blotting	Reduced expression: MITF, TYR, TYRP-1, TYRP-2	[31]
*Dendropanax morbiferus* H.Lév./Araliaceae	leaves	ethanol	12.5–50 µg/mL	Western blotting	Reduced expression: TYR, TYRP-2	[32]
*Equisetum ramosissimum* Desf./Equisetaceae	whole plant	ethyl acetate, dichloromethane	10–100 µg/mL	Western blotting Western blotting	ethyl acetate: Reduced expression: MITF, TYR, TYRP-1, TYRP-2; dichloromethane: Increased expression: MITF, TYR, TYRP-1, TYRP-2	[33]
*Euryale ferox* Salisb./Nymphaeaceae	seeds	ethyl acetate	30 µg/mL	RT-PCR, Western blotting	Reduced expression: MITF, TYR, TYRP-1, TYRP-2	[34]
*Gaillardia aristata* Pursh/Asteraceae	flowers	ethanol	10–20 µg/mL	RT-PCR, Western blotting	Reduced expression: MITF, TYR, TYRP-1, TYRP-2	[35]
*Garcinia mangostana* L./Clusiaceae	leaves	water	4–32 µg/mL	Western blotting	Increased expression: TYR	[36]
*Gastrodia elata* Blume/Orchidaceae	whole plant	water	0.5–5 mg/mL	RT-PCR, Western blotting	Reduced expression: MITF, TYR, TYRP-1, TYRP-2	[37]
*Glechoma hederacea* L./Lamiaceae	whole plant	water	0.1–1 mg/mL	RT-PCR, Western blotting	Reduced expression: TYR	[38]
Glycine max (L.) Merr./Fabaceae	seeds cell culture	ethanol	0.5–1 mg/mL	RT-PCR, Western blotting	Reduced expression: MITF, TYR, TYRP-1, TYRP-2	[39]
*Haloxylon scoparium* Pomel/Amaranthaceae	stems	ethanol	0.017%(*w*/*v*)	RT-PCR, Western blotting	Reduced expression: MC1R, TYR, TYRP-1	[40]
*Kummerowia striata* (Thunb.) Schindl./Fabaceae	aerial parts	ethanol	100–400 µg/mL	RT-PCR, Western blotting	Reduced expression: MITF, TYR, TYRP-1, TYRP-2	[41]
*Kummerowia striata* (Thunb.) Schindl./Fabaceae	aerial parts	ethanol	100–400 µg/mL	RT-PCR, Western blotting	Reduced expression: MITF, TYR, TYRP-1, TYRP-2	[42]
*Melia azedarach* L./Meliaceae	whole plant	ethanol	20 µg/mL	RT-PCR, Western blotting	Increased expression: TYRP-1	[43]
*Nepeta binaludensis* Jamzad/Lamiaceae	aerial parts	methanol	50 µg/mL	Western blotting	Reduced expression: TYR	[44]
*Nepeta sintenisii* Bornm./Lamiaceae	aerial parts	n-hexane, methanol, water	50 µg/mL	Western blotting	Reduced expression: MITF	[45]
*Oplismenus undulatifolius* (Ard.) P.Beauv./Poaceae	whole plant	ethanol	5–15 µg/mL	Western blotting	Reduced expression: TYR, TYRP-1, TYRP-2	[46]
*Oreocnide fruticosa* (Gaudich.) Hand. Mazz./Urticaceae	branches	ethyl acetate	25–100 µg/mL	Western blotting	Reduced expression: TYR, TYRP-1, TYRP-2	[47]
*Phyllanthus emblica* L./Phyllanthaceae	fruits	water	0.05–1 mg/mL	RT-PCR, Western blotting	Reduced expression: MITF, TYR, TYRP-1, TYRP-2	[48]
*Pinus densiflora* Siebold & Zucc./Pinaceae	pine cone	ethyl acetate	12.5–50 µg/mL	RT-PCR, Western blotting	Reduced expression: MITF, TYR, TYRP-1, TYRP-2	[49]
*Psoralea corylifolia* (Babchi)/Fabaceae and *Zingiber officinale* Roscoe/Zingiberaceae; *Psoralea corylifolia* (Babchi)/Fabaceae and *Eclipta prostrata* (L.) L./Asteraceae	whole plants	methanol	10–100 µg/mL	RT-PCR, Western blotting	Increased expression: MITF	[50]
*Syzygium cumini* (L.) Skeels/Myrtaceae	leaves and branch	ethanol	25–100 µg/mL	RT-PCR	Reduced expression: TYR, TYRP-1, TYRP-2	[51]
*Uncaria rhynchophylla* (Miq.) Miq./Rubiaceae	stems and hooks	ethanol	0.1–1 mg/mL	RT-PCR	Reduced expression: TYR	[52]
*Vitis vinifera* L./Vitaceae	pericarp, seed, flesh, and grape stem	ethanol	100 µg/mL	Western blotting	Increased expression: MITF, TYR, TYRP-1, TYRP-2	[53]

**Table 2:** Modulation on gene expression related to melanogenesis in B16 melanoma cells by plant extracts with identified compounds.

Name of Species/Family	Part of Plant	Type of Solvent	Identified Compounds	Concentration	Methods	Effects	Ref.
*Acer rubrum* L./Sapindaceae	leaves	ethanol	phenolic compounds	10 µg/mL	RT-PCR, Western blotting	Reduced expression: MITF, TYR, TYRP-1, TYRP-2	[54]
*Angelica polymorpha* Maxim./Apiaceae	flowers	hexane	aromadendrene, methoxsalen, bergapten, isopimpinellin, nonadencane	0.1–100 µg/mL	Western blotting	Reduced expression: MITF, TYR	[55]
*Argania spinosa* L.) Skeels/Sapotaceae	leaves	ethanol	14 compounds	30 µg/mL	Western blotting	Increased expression: TYR, TYRP-1,	[56]
*Artemisia capillaris* Thunb./Asteraceae	whole plant	ethanol	leukodin	12.5–50 µg/mL	Western blotting	Reduced expression: TYR, TYRP-1, TYRP-2	[57]
*Artocarpus lacucha* Buch.-Ham./Moraceae and Glycyrrhiza glabra L./Fabaceae	heartwood and root	ethanol	gallic acid, oxyresveratrol, resveratrol and glabridin	0.1 mg/mL	Western blotting	Reduced expression: MITF, TYRP-2	[58]
*Callicarpa longissima* (Hemsl.) Merr./Lamiaceae	whole plant	ethanol	carnosol and carnosic acid	0.1–10 µg/mL	RT-PCR	Reduced expression: MITF	[59]
*Ceratonia siliqua* L./Fabaceae	leaves, bark and fruits	ethanol	epicatechin-3-*O*-gallate, 1,2,3,6-tetra-*O*-galloyl-ß-D-glucose and gallocatechin-3-*O*-gallate	100 µg/mL	RT-PCR	Reduced expression: TYR	[60]
*Glycyrrhiza glabra* L. and Glycyrrhiza uralensis Fisch. ex DC./Fabaceae	whole plant/heat treated	ethanol	isoliquiritigenin	100 µg/mL	RT-PCR, Western blotting	Reduced expression: MITF, TYR, TYRP-1, TYRP-2	[61]
*Hordeum vulgare* L./Poaceae	barely sprout	water	p-coumaric, ferulic, and vanillic acids	50–250 µg/mL	Western blotting	Reduced expression: MITF, TYR	[62]
*Juniperus communis* L./Cupressaceae	fruits	ethanol	hypolaetin-7-*O*-β-D-xylopyranoside and isoscutellarein-7-*O*-β-D-xylopyranoside	50 µg/mL	Western blotting	Reduced expression: TYR	[63]
*Libidibia ferrea* (Mart. ex Tul.) L.P.Queiroz/Fabaceae	bark and pods	ethanol	18 compounds	25 µg/mL	RT-PCR, Western blotting	Reduced expression: TYR	[64]
*Limonium tetragonum* (Thunb.) Bullock/Plumbaginaceae	whole plant	water, methanol, buthanol	myricetin 3-galactoside and quercetin 3-O- -galactopyronaside	5–20 µg/mL	RT-PCR, Western blotting	Reduced expression: MITF, TYR, TYRP-1, TYRP-2	[65]
*Myrica rubra* (Lour.) Siebold & Zucc./Myricaceae	fruits	water	myricetin-*O*-deoxyhexoside, quercetin-*O*-deoxyhexoside, and aempferol-O-hexoside	0.5–2 mg/mL	RT-PCR, Western blotting	Reduced expression: MITF, TYRP-1,	[66]
*Nigella sativa* L./Ranunculaceae	seed	Thymocid^®^	thymoquinone	20 µg/mL	RT-PCR, Western blotting	Reduced expression: MITF, TYRP-1, TYRP-2	[67]
*Petasites japonicus* (Siebold & Zucc.) Maxim./Asteraceae	leaves, stems, and roots	water	leaf extract-isorhamnetin (main) root extract-p-coumaric acid (main)	50–200 µg/mL	RT-PCR, Western blotting	Reduced expression: TYR	[68]
*Phyllanthus emblica* L./Phyllanthaceae	branch	ethanol	gallic acid and vanillic acid	6.25–25 µg/mL	RT-PCR	Reduced expression: TYR, TYRP-1, TYRP-2	[69]
*Pueraria montana* (Lour.) Merr./Fabaceae	aerial parts	ethanol	daidzein, daidzin, glycitein, glycitin, genistein, genistin	10–100 µg/mL	RT-PCR, Western blotting	Reduced expression: TYR	[70]
*Pueraria montana* (Lour.) Merr./Fabaceae	stems	n-hexane	12 compounds	50 µg/mL	RT-PCR	Reduced expression: TYR	[71]
*Rhododendron weyrichii* Maxim./Ericaceae Durande	flowers	ethanol	p-coumaric acid	25–200 µg/mL	Western blotting	Reduced expression: TYR, TYRP-1, TYRP-2	[72]
*Sorghum bicolor* (L.) Moench/Poaceae	whole plant	ethanol	1-*O*-ca eoylglycerol, dica eoylglycerides, 1,3-*O*-dica eoylglycerol, p-coumaroyl-ca eoylglycerol, feruloyl-ca eoylglycerol, Tricin, 9-hydroxyoctadecadienoic acid	2–10 µg/mL	Western blotting	Reduced expression: MITF, TYRP-1,	[73]
*Vernonia anthelmintica* (L.) Willd./Asteraceae	whole plant	ethanol	15 compounds (mainly flavonoids)	20 µg/mL	Western blotting	Increased expression: TYR	[74]

**Table 3:** Modulation on gene expression related to melanogenesis in B16 melanoma cells by isolated plant compounds.

Name of Species/Family	Part of Plant	Compounds	Concentration	Methods	Effects	Ref.
*Acanthopanax koreanum* Nakai/Araliaceae	roots	acanthoic acid	25–100 µg/mL	Western blotting	Reduced expression: TYR, TYRP-1, TYRP-2	[75]
*Artemisia capillaris* Thunb./Asteraceae	whole plant	leukodin	37.5–150 µg/mL	Western blotting	Reduced expression: TYRP-1, TYRP-2	[57]
*Artemisia capillaris* Thunb./Asteraceae	whole plant	isofraxidin 7-*O*-(6′-*O*-p-coumaroyl)-𝛽-glucopyranoside	25 µg/mL	RT-PCR	Increased expression: MITF, TYR	[76]
*Artemisia capillaris* Thunb./Asteraceae	leaves and stems	4,5-𝑂-dicaffeoylquinic acid	25 µg/mL	RT-PCR	Reduced expression: TYRP-1	[77]
*Caesalpinia sappan* L./Fabaceae	heartwood	sappanone A	4.4 µg/mL	RT-PCR	Reduced expression: TYR	[78]
*Crocus sativus* L./Iridaceae	stigmas	crocetin	0.5–32 µg/mL	Western blotting	Reduced expression: MITF	[79]
*Cuscuta chinensis* Lam./Convolvulaceae	whole plant	polysaccharide	40–160 µg/mL	Western blotting	Reduced expression: MITF, TYR, TYRP-1	[80]
*Ephedra sinica* Stapf/Ephedraceae	roots	ephedrannins A and B	A: 18–72 µg/mL; B: 1.85–7.4 µg/mL	RT-PCR	Reduced expression: TYR	[81]
*Fragaria × ananassa* (Duchesne ex Weston) Duchesne ex Rozier/Rosaceae	calyx	oleanolic acid	12.5 µg/mL	Western blotting	Reduced expression: TYR, TYRP-1, TYRP-2	[82]
*Isodon trichocarpus* (Maxim.) Kudô./Lamiaceae	aerial parts	enmein, isodocarpin, nodosin, oridonin	1–3 µg/mL	RT-PCR	Reduced expression: TYR, TYRP-1, TYRP-2	[83]
*Jatropha multifida* L./Euphorbiaceae	stems	Secoisolariciresinol	6.25–200 µg/mL	RT-PCR	Reduced expression: TYR	[84]
*Kaempferia parviflora* Wall. ex Baker/Zingiberaceae	rhizomes	5-hydroxy-7,3′,4′-trimethoxyflavone, 5,7,3′,4′-tetramethoxyflavone, 5,3′- dihydroxy-3,7,4′-trimethoxyflavone and 5-hydroxy-3,7,3′,4′-tetramethoxyflavone	3–30 µg/mL	RT-PCR	Reduced expression: TYR, TYRP-1, TYRP-2	[85]
*Limonium tetragonum* (Thunb.) Bullock/Plumbaginaceae	whole plant	myricetin 3-galactoside and quercetin 3-O-galactopyronaside	10 µg/mL	Western blotting	Reduced expression: TYRP-1, TYRP-2	[65]
*Persicaria amphibia* (L.) Delarbre/Polygonaceae	whole plant	epicatechin-3-*O*-gallate	25–200 µg/mL	Western blotting	Reduced expression: MITF, TYR, TYRP-1, TYRP-2	[86]
*Pteris dispar* Kunze/Pteridaceae	leaves	ent -11α-hydroxy-15-oxo-kaur-16-en-19-oic acid	10 µg/mL	RT-PCR, Western blotting	Reduced expression: TYR	[87]
*Pyracantha angustifolia* (Franch.) C.K.Schneid./Rosaceae	leaves, twigs, and fruits	β-D-glucosylester and cimidahurinine	10–100 µg/mL	Western blotting	Reduced expression: TYRP-1, TYRP-2	[88]
*Stewartia pseudocamellia* Maxim.	twigs	stewartianol and stewartianol- 3-*O*-glucoside	20–80 µg/mL	Western blotting	Reduced expression: MITF	[89]
*Tetragonia tetragonoides* (Pall.) Kuntze/Aizoaceae	whole plant	ferulic acid	5–20 µg/mL	Western blotting	Reduced expression: MITF, TYR	[90]
*Vitellaria paradoxa* C.F.Gaertn./Sapotaceae	fruit	glucosylcucurbic acid and cucurbic acid	30–100 µg/mL	Western blotting	Reduced expression: MITF, TYR, TYRP-1, TYRP-2	[91]
*Weigela subsessilis* (Nakai) L.H.Bailey/Caprifoliaceae	aerial parts	loniceroside A, loniceroside L	1–20 µg/mL	Western blotting	Increased expression: MITF, TYR	[92]

## Data Availability

Not applicable.

## Sample Availability

Samples of the compounds are available from the authors.
